# Attitudes towards the economic costs associated with measures against the spread of COVID-19: Population perceptions from repeated cross-sectional data of the nationally representative COVID-19 Snapshot Monitoring in Germany (COSMO)

**DOI:** 10.1371/journal.pone.0259451

**Published:** 2021-11-05

**Authors:** André Hajek, Freia De Bock, Philipp Sprengholz, Benedikt Kretzler, Hans-Helmut König

**Affiliations:** 1 Department of Health Economics and Health Services Research, Hamburg Center for Health Economics, University Medical Center Hamburg-Eppendorf, Hamburg, Germany; 2 Federal Centre for Health Education, Köln, Germany; 3 Department of Health Communication, University of Erfurt, Erfurt, Germany; Flinders University School of Medicine: Flinders University Medicine, AUSTRALIA

## Abstract

**Introduction:**

Our aim was to examine attitudes of the general population towards reasonableness of these costs, as well as the degree to which these costs are shared across society (solidarity financing) and to determine the factors associated with them.

**Method:**

Repeated cross-sectional data from a nationally representative online-survey. More precisely, data from wave 8 (21–22 April 2020) and wave 16 (7–8 July 2020) were used (in wave 8: analytical sample with n = 976, average age was 47.0 years (SD: 15.3 years), ranging from 18 to 74 years, 51.8% female; in wave 16: analytical sample with n = 978, average age was 46.1 years (SD: 15.9 years), ranging from 18 to 74 years, 50.9% female). After a short introduction emphasizing considerable economic costs associated with the measures against the spread of the coronavirus, individuals were asked to rate the following statements (outcome measures), in each case from 1 = strongly disagree to 7 = strongly agree: “These economic costs are currently reasonable in relation to the objective pursued” (reasonableness of costs), “These economic costs should be borne jointly by all citizens and depending on income” (solidarity financing).

**Results:**

In wave 8 (wave 16 in parentheses), the average rating for the attitude towards reasonableness of costs was 4.3, SD: 1.8 (wave 16, average: 4.2, SD: 1.8) and the average rating for the attitude towards solidarity financing was 3.7, SD: 1.9 (wave 16, average: 3.3, SD: 2.0). In wave 8, more positive attitudes towards the reasonableness of costs and solidarity financing were associated with being male, higher education, not being in a partnership/being unmarried, higher affect regarding COVID-19 and higher presumed severity with respect to COVID-19. Furthermore, more positive attitudes towards the reasonableness of costs were associated with having a migration background. More positive attitudes towards solidarity financing was associated with higher age groups. Mainly similar findings were observed in wave 16.

**Discussion:**

Agreement with reasonableness of costs of preventative measures as well as solidarity financing was moderately high. Knowledge of these attitudes is important to ensure social cohesion during the fight against COVID-19.

## 1. Introduction

The adequacy of measures to protect health during the COVID-19 pandemic will ultimately be assessed based on their direct positive, but also negative effects on health, social life and the economy [1,2]. Specifically, (near) lock-down measures (further details regarding the term lockdown are presented elsewhere [3]) have to be balanced out with their potential negative effects such as economic costs, as becomes currently visible by changing tones in the debate about potential new school closures in the autumn of 2020 [4].

Knowledge about how the general population perceives the activities implemented to prevent the spread of COVID-19 is important to prepare for communication about costs and hardships.

It is also important to understand the population’s perception on how these activities should be financed, namely whether the costs should be shared across society.

In Germany, like in other countries, discussion about the economic costs of the lock-down policies has been part of the public debate from the start on. According to the ifo Institute the German economy will probably shrink by 6.6 percent in 2020 [5]. If these predictions come true, this would be the largest shrinkage in the gross domestic product since the 1950s (except for the global financial and economic crisis of 2008/2009 where the gross domestic product decreased by about 5.8%). Similar projections have been reported in other countries [6].

Thus far, there is a general lack of studies focusing on the attitudes of the general population towards reasonableness of these costs, as well as the degree to which these costs are shared across society (solidarity financing). One recent study [7] focused on the first week of lockdown in Germany. Tepe et al. [7] performed two survey experiments focusing on how the COVID-19 lockdown affects how individuals in Germany trade off lives and weigh constitutional powers. Individuals were called to think about the COVID-19 lockdown in a priming experiment. They found no change in ‘federal vs. state’ power balance preferences, whereas they found an increase in support for changing power from parliaments to governments [7]. However, the existing studies did not explicitly focus on the attitudes of the general population towards reasonableness of these costs and towards solidarity financing. Thus, in view of the lack of corresponding data on attitudes of the general population towards reasonableness of these costs and towards solidarity financing, this work aims to examine attitudes of the general population towards reasonableness of these costs, as well as the degree to which these costs are shared across society (solidarity financing) and to examine the correlates in Germany. As our study was exploratory, we did not have any specific hypotheses regarding the correlates. We used repeated cross-sectional data to identify potential changes in the outcomes from April to July 2020. We assume rather high agreement at the beginning of the pandemic (in a state of shock), which then tends to decrease over time (with lower incidence).

It should be repeated that this knowledge is important as it provides a rough approximation of the acceptance of such activities in society in April (two days after end of first lockdown) and July 2020 (no lockdown, few restrictions). Ultimately, this is important to maintain social cohesion within countries.

In Germany, nationwide COVID-19-measures were implemented on 16^th^ March 2020. These measures covered, among other things, closure of schools and day-care centers. These measures were intensified on 22^nd^ March 2020 by implementing travel bans or contact restrictions in public. In the upcoming weeks, prolongations of these measures took place. On 20^th^ April 2020, some COVID-19 measures were loosened such as shops under a certain size were allowed to reopen. By early May (4^th^ May), schools slowly started to open again. Further loosening of the restrictions took place in May such as loosening the contact ban or reopening museums or playgrounds. Restrictions were further eased in June and remained relatively stable in July 2020. However, in case the number of infections increases, an “emergency brake” was implemented (i.e., if the rate locally exceeds 50 diagnoses per 100,000 inhabitants over a seven-day period, the restrictions could be tightened again locally).

## 2. Materials and methods

### 2.1 Sample

Repeated cross-sectional data were derived from wave 8 and wave 16 of the COVID-19 Snapshot Monitoring (COSMO) [8]. Exclusively these two waves were used due to reasons of data availability. More precisely, the outcome measures were exclusively assessed in these waves.

The COSMO study started in March 2020 (3^rd^ to 4^th^ March 2020) with weekly cross-sectional online-surveys (15–20 minutes) on the COVID-19-situation for the general public. In each wave around 1,000 respondents complete an online questionnaire. The eighth wave took place from 21^st^ to 22^nd^ April 2020 (wave 16: 7^th^ to 8^th^ July 2020). The individuals were recruited from a market research company named Respondi (https://www.respondi.com/ - ISO 26362 certified online sample provider).

Sampling was quota-based: Individuals were drawn from a pool of respondents (so-called online panel) in such a way that it corresponds to the distribution of age, gender (crossed-quota: gender x age) and federal state (uncrossed) in the German population [9]. Both, wave 8 and wave 16 included German-speaking individuals aged 18 to 74 years living in Germany. This means that younger (17 years or younger) and older individuals (75 years and over) were not included in wave 8 and wave 16 of the COSMO study. Respondents were compensated for participation via Respondi at their usual rate. All individuals between 18 and 74 years of age completing the survey were eligible for inclusion into the analyses and comprise the final sample.

Repeated cross-sectional data were used to display potential trends over time (i.e., from April to July 2020) in the outcome measures. However, it should be emphasized that it is impossible to examine individual trajectories over time when using repeated cross-sectional data.

Informed consent was obtained from all individual participants included in the study. Ethical approval for COSMO was obtained by University of Erfurt’s IRB (#202000302). All procedures performed in the COSMO studies involving human participants were in accordance with the ethical standards of the University of Erfurt institutional research committee and with the 1964 Helsinki Declaration and its later amendments or comparable ethical standards.

The data from the COVID-19 Snapshot Monitoring (COSMO) are stored in the repository PsychArchives (https://doi.org/10.23668/psycharchives.2776) and are made available to scientists upon request. Requests should be submitted to the COSMO consortium by contacting Prof. Dr. Cornelia Betsch, University of Erfurt, Nordhäuser Str. 63, 99089 Erfurt, Germany (cornelia.betsch@unierfurt.de).

### 2.2 Dependent variables

After a short introduction (“Economic experts have calculated that the measures against the spread of the coronavirus (e.g. closure of businesses, ban on events) will cause considerable economic costs”), individuals were asked to rate the following statements (our outcome measures), in each case from 1 = strongly disagree to 7 = strongly agree (in both cases, only these endpoints were labeled):

- “These economic costs are currently reasonable in relation to the objective pursued” (reasonableness of costs)
- “These economic costs should be borne jointly by all citizens and depending on income” (solidarity financing)

To reduce information bias, a small pretest was conducted (with n = 15) which confirmed the comprehensibility and face validity of our dependent variables.

### 2.3 Independent variables

In regression analysis, we included sociodemographic factors and factors related to COVID-19 because we were interested in the fact whether such factors are associated with both outcome measures. More precisely, the following sociodemographic independent variables were included: sex (women; men), age category (18 to 29 years; 30 to 49 years; 50 to 64 years; 65 years and over), children under 18 years (no; yes), relationship/marriage (no; yes), living situation (living alone; at least two individuals in the same household), education (up to 9 years / 10 years and more (without general qualification for university entrance); 10 years and more (with general qualification for university entrance)), migration background (no; yes), self-employed (no; yes), chronic diseases (no; yes), region (West Germany (i.e., federal states which belonged to former West Germany); East Germany (i.e., federal states which belonged to former East Germany)), town size (municipality/small town (1–20.000); medium sized town (20.001–100.000); small city (100.001–500.000); big city (> 500.000)) and the COVID-19 cases/100,000 population (below median; above median).

Furthermore, affect (such as fear or concerns) regarding COVID-19 (from 1 to 7; higher values correspond to higher affect) and presumed severity of COVID-19 infection (from 1 to 7; higher values correspond to higher severity) were used as independent variables. Exemplary items for the tool quantifying affect regarding COVID-19 (consisting of seven items as listed in S1 Table), were: For me, the new type of coronavirus is… “near” (1) to “far away” (7), or “scary” (1) to “not scary” (7). As shown in S1 Table, five items were recoded. Subsequently, items were averaged to create the final score. In our study, Cronbach’s alpha was .78 (wave 8) and .80 (wave 16). As regards presumed severity of COVID-19 infection, the exact wording was: “How do you assess an infection with the novel coronavirus for yourself?” (from 1 = completely harmless to 7 = extremely dangerous).

### 2.4 Statistical analysis

Sample characteristics for our analytical sample were first calculated. We also calculated the effect size (Cohen’s d) to assess the size of potential differences in the outcomes from wave 8 to wave 16. Subsequently, four multiple linear regressions were used to determine the factors associated with both outcome measures (both waves separately).

We performed a White test for heteroscedasticity in the error distribution. According to the test statistics (e.g., in wave 16 with reasonableness of costs as outcome measure, White’s general test statistic equaled 219.28, p < 0.01; with solidarity financing as outcome measure, White’ general test statistic equaled 256.27, p < .001), residuals are heteroscedastic. Therefore, robust standard errors were calculated in this study. Additionally, standardized normal probability plots were used to check the normality of residuals. According to these plots, the residuals have an approximately normal distribution. We also checked for multicollinearity. The variance inflation factors (VIFs) were very low. This indicates that multicollinearity is not a threat.

The statistical significance was defined as p value of ≤ .05. Statistical analyses were performed using Stata 16.1 (Stata Corp., College Station, Texas).

## 3. Results

### 3.1 Sample characteristics

**Fig 1:**
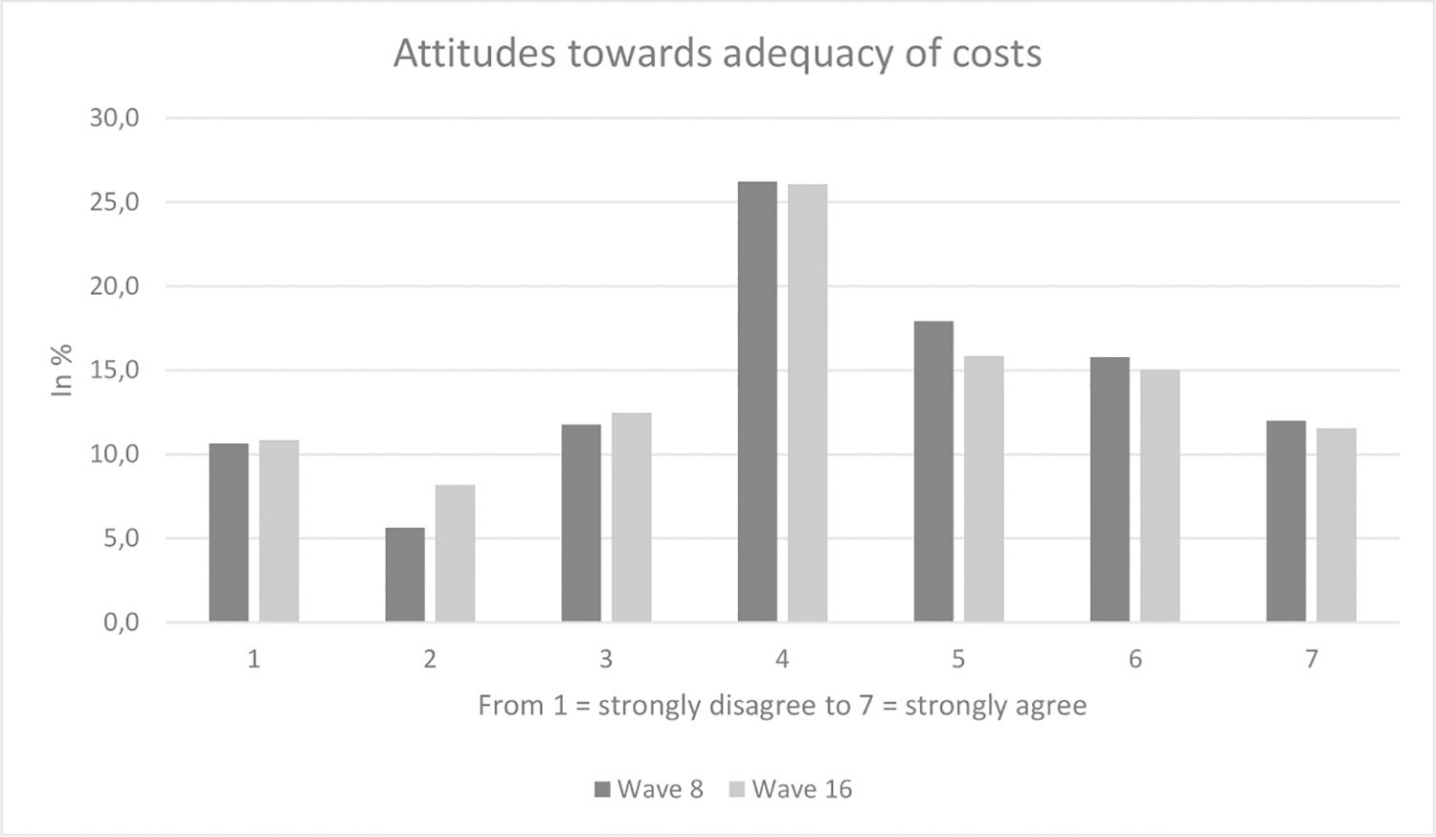
Attitudes towards reasonableness of costs.

**Fig 2:**
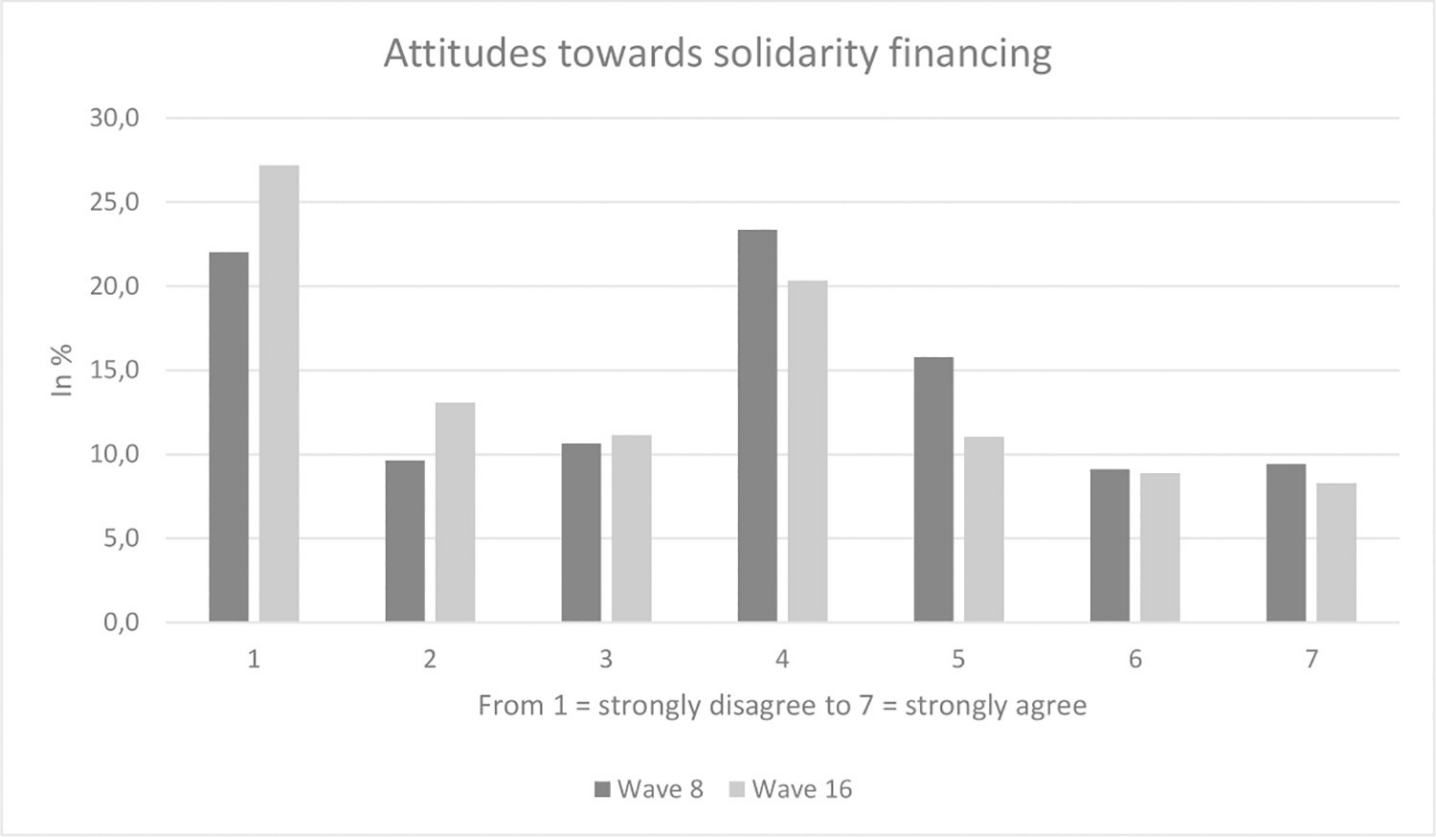
Attitudes towards solidarity financing.

Sample characteristics for our analytical sample (wave 8; n = 976 out of 1,014 due to a few missing values in the independent variables) are displayed in Table 1. Average age equaled 47.0 years (SD: 15.3 years), ranging from 18 to 74 years. 51.8% of the participants were female and 56.1% had 10 years or more of education (with general qualification for university entrance). The average rating for attitudes towards reasonableness of costs was 4.3 (SD: 1.8, ranging from 1 to 7) and the average rating for the attitude towards solidarity financing was 3.7 (SD: 1.9, ranging from 1 to 7). Sample characteristics were similar for wave 16. The average rating for attitudes towards reasonableness of costs was 4.2 (SD: 1.8, ranging from 1 to 7) and the average rating for the attitude towards solidarity financing was 3.3 (SD: 2.0, ranging from 1 to 7). Furthermore, two histograms (Figs 1 and 2) provide a graphical picture of our outcome measures. According to Fig 2, there is a bimodal distribution in attitudes towards solidarity financing.

**Table 1:** Sample characteristics for the analytical sample (n = 976 individuals) at wave 8.

	Mean (SD) / n (%)
Sex	
Men	470 (48.2%)
Women	506 (51.8%)
Age category	
18 to 29 years	159 (16.3%)
30 to 49 years	378 (38.7%)
50 to 64 years	289 (29.6%)
65 years and over	150 (15.4%)
Children under 18 years:	
No	716 (73.4%)
Yes	260 (26.6%)
Education	
up to 9 years / 10 years and more (without general qualification for university entrance)	428 (43.9%)
10 years and more (with general qualification for university entrance)	548 (56.1%)
Town size	
Municipality/small town (1–20.000)	369 (37.8%)
Medium sized town (20.001–100.000)	230 (23.6%)
Small city (100.001–500.000)	176 (18.0%)
Big city (> 500.000)	201 (20.6%)
Region	
West Germany	833 (85.3%)
East Germany	143 (14.7%)
Cases/100,000 population	
Below median	450 (46.1%)
Above median	526 (53.9%)
Relationship/Marriage	
No	300 (30.7%)
Yes	676 (69.3%)
Living situation	
Living alone	244 (25.0%)
At least 2 individuals in the same household	732 (75.0%)
Migration background:	
No	840 (86.1%)
Yes	136 (13.9%)
Self-employment	
No	880 (90.2%)
Yes	96 (9.8%)
Chronic disease	
No	633 (64.9%)
Yes	343 (35.1%)
Affect: COVID-19 infection (from 1 to 7; higher values correspond to higher affect)	4.3 (1.0)
Severity: COVID-19 infection (from 1 to 7; higher values correspond to higher severity)	4.0 (1.5)

The effect size (1. comparing the average rating for the attitude towards reasonableness of costs at wave 8 with the corresponding average rating at wave 16; 2. comparing the average rating for the attitude towards solidarity financing at wave 8 with the corresponding average rating at wave 16)) were in the first case Cohen’s d = -0.04 and in the second case Cohen’s d = -0.21.

### 3.2 Regression analysis

Multiple linear regressions with attitudes towards reasonableness of costs (wave 8: column 2, wave 16: column 4) and solidarity financing (wave 8: column 3, wave 16: column 5) are displayed in Table 2. In other words, four multiple linear regression models were estimated:

1. Second column: With attitudes towards reasonableness of costs as outcome measure (wave 8)
2. Third column: With attitudes towards solidarity financing as outcome measure (wave 8)
3. Fourth column: With attitudes towards reasonableness of costs as outcome measure (wave 16)
4. Fifth column: With attitudes towards solidarity financing as outcome measure (wave 16)

**Table 2:** Determinants of attitudes towards reasonableness of economic costs (column 2 and column 4) and solidarity financing (column 3 and column 5). Results of multiple linear regressions.

	Wave 8		Wave 16	
Independent variables	Attitudes towards reasonableness of costs	Attitudes towards solidarity financing	Attitudes towards reasonableness of costs	Attitudes towards solidarity financing
Gender: Female (Ref.: Male)	-0.27*	-0.52***	-0.23*	-0.56***
	(0.11)	(0.12)	(0.11)	(0.12)
Age category: - 30 to 49 years (Ref.: 18 to 29 years)	-0.19	0.06	-0.15	-0.14
	(0.16)	(0.18)	(0.16)	(0.18)
- 50 to 64 years	0.13	0.40*	-0.12	-0.14
	(0.18)	(0.20)	(0.18)	(0.20)
- 65 years and over	0.18	0.57*	-0.12	0.15
	(0.19)	(0.23)	(0.21)	(0.23)
Children (under 18 years): Yes (Ref.: Absence of children under 18 years)	-0.05	0.22	-0.25+	0.05
	(0.14)	(0.16)	(0.15)	(0.16)
Education: General qualification for university entrance (Ref.: absence of qualification for university entrance)	0.25*	0.30*	0.09	0.27*
	(0.12)	(0.13)	(0.11)	(0.13)
Town size:—Medium sized town (20.001–100.000) (Ref.: municipality/small town (1–20.000))	-0.25+	-0.01	0.21	0.14
	(0.14)	(0.16)	(0.14)	(0.15)
- Small city (100.001–500.000)	0.14	0.11	0.29+	0.21
	(0.16)	(0.18)	(0.16)	(0.18)
- Big city (> 500.000)	-0.18	0.05	-0.06	0.33+
	(0.15)	(0.17)	(0.16)	(0.18)
Region: East Germany (Ref.: West Germany)	0.04	-0.13	-0.06	-0.48**
	(0.17)	(0.19)	(0.17)	(0.18)
Cases/100,000 population: Above median (Ref.: below median)	0.09	-0.04	0.01	-0.16
	(0.12)	(0.13)	(0.13)	(0.15)
Relationship/Marriage: Yes (Ref.: no partnership/marriage)	-0.34*	-0.37*	-0.01	-0.21
	(0.15)	(0.18)	(0.15)	(0.17)
Living situation: At least 2 individuals in the same household (Ref.: living alone)	0.14	0.12	-0.05	0.08
	(0.17)	(0.19)	(0.17)	(0.20)
Migration background: Yes (Ref.: no migration background)	0.37*	0.12	-0.09	0.04
	(0.14)	(0.18)	(0.15)	(0.17)
Self-employment: Yes (Ref.: not self-employed)	-0.06	-0.15	0.36+	0.22
	(0.18)	(0.22)	(0.20)	(0.25)
Chronic disease: Yes (Ref.: no chronic diseases)	-0.07	0.03	-0.31*	-0.11
	(0.12)	(0.14)	(0.12)	(0.14)
Affect: COVID-19 infection (higher values correspond to higher affect)	0.52***	0.19**	0.40***	0.22**
	(0.06)	(0.07)	(0.07)	(0.07)
Severity: COVID-19 infection (higher values correspond to higher severity)	0.11*	0.12*	0.17***	0.16**
	(0.04)	(0.05)	(0.05)	(0.05)
Constant	1.48***	2.20***	2.09***	2.31***
	(0.43)	(0.48)	(0.45)	(0.48)
Observations	976	976	978	978
R^2^	0.15	0.06	0.14	0.09

Unstandardized beta-coefficients are reported; robust standard errors in parentheses

*** p<0.001

** p<0.01

* p<0.05, + p<0.10. The outcome measures were assessed as follows (in each case from 1 = strongly disagree to 7 = strongly agree): “These economic costs are currently reasonable in relation to the objective pursued.” (attitudes towards reasonableness of costs); “These economic costs should be borne jointly by all citizens and depending on income.” (attitudes towards solidarity financing).

Listwise deletion was used to handle missing values.

With regard to wave 8, regressions showed that stronger agreement with the statements that the economic costs are reasonable and that these costs should be borne by all citizens was associated with being male (with attitudes towards reasonableness of costs: β = -.27, p < .05; with attitudes towards solidarity financing: β = -.52, p < .001), higher education (β = .25, p < .05; β = .30, p < .05), not being in a partnership/being unmarried (β = -.34, p < .05; β = -.37, p < .05), higher affect regarding COVID-19 (β = .52, p < .001; β = .19, p < .01) and higher presumed severity of COVID-19 (β = .11, p < .05; β = .12, p < .05). Furthermore, more positive attitudes towards the reasonableness of costs were associated with having a migration background (β = .37, p < .05). More positive attitudes towards solidarity financing were associated with higher age groups (for example: 65 years and over: β = .57, p < .05).

While various associations remained remarkably similar in wave 16, some differences are worth noting: In wave 16, the link between higher age groups, and attitudes towards solidarity financing disappeared. Furthermore, more negative attitudes towards solidarity financing were present in East Germany (β = -.48, p < .01). Moreover, the link between migration background and attitudes towards reasonableness of costs vanished. Additionally, the association between chronic diseases and attitudes towards reasonableness of costs became significant (β = -.31, p < .05). Furthermore, the association between relationship status and both outcome measures disappeared.

## 4. Discussion

### 4.1 Main findings

Our aim was to descriptively analyze attitudes towards the economic costs associated with measures against the spread of COVID-19, and to determine the factors associated with them. Agreement with reasonableness of costs as well as solidarity financing was moderately high. We could not identify a substantial change over time in both outcome measures. Several sociodemographic and COVID-19-related correlates have been identified.

### 4.2 Previous research and possible explanations

It should be noted that there is a general lack of studies investigating the attitudes towards reasonableness of these costs, as well as the degree to which these costs are shared across society. Therefore, our findings are difficult to compare with other studies. Nevertheless, it appears plausible that attitudes towards the reasonableness of costs and solidarity financing were positively associated with higher affect regarding COVID-19 and higher presumed severity of COVID-19. Potential individual health consequences may increase the desire that society as a whole covers the costs associated with the COVID-19 pandemic. Furthermore, when individuals fear the severity of a COVID-19 infection, they may be more willing to accept the restrictions in daily life, and ultimately may appreciate the actions taken to address this pandemic. Similarly, the link between higher age and more positive attitudes towards solidarity financing can be explained by potential individual health consequences. However, it should be noted that this link disappeared in wave 16.

Higher education may reflect a higher ability to pay and may also reflect the increased ability to critically reflect on the pandemic and its economic consequences [10]. Moreover, higher education may reflect an increased perception of the necessity of these actions. Therefore, individuals with high education may report more positive attitudes towards the associated costs and solidarity financing. However, future studies are necessary to test our possible explanations.

Surprisingly, unmarried/single and male individuals reported more positive attitudes towards the reasonableness of costs and solidarity financing. However, the association between marital status and the outcomes vanished in wave 16. In addition, more positive attitudes towards the reasonableness of the costs were associated with having a migration background. Again, this association vanished in wave 16. Future research is required to clarify this association in further detail.

Interestingly, the agreement with reasonableness of costs as well as solidarity financing was moderately high in both waves (worth repeating, wave 8: 21–22 April 2020; wave 16: 7–8 July 2020). This means that individuals reported similar agreements when the partial lockdown with restrictions of social gatherings, closing of schools, universities, cultural events and restaurants was first eased in April and in July (when various restrictions were further eased). Future research in the next months is required to better understand these agreements. Moreover, qualitative studies are desirable to gain deeper insights into these agreements.

It should be repeated that we could not determine a considerable change over time in both outcomes. This appears somewhat surprising given the fact that wave 8 took place two days after the first lockdown and wave 16 took place in a time with few restrictions. A possible explanation may be that our outcomes reflect more stable constructs rather than quickly changing attitudes. However, future research is required to investigate these outcomes in later stages of the COVID-19 pandemic.

### 4.3 Strengths and limitations

Some strengths and limitations of this study are worth highlighting. To the best of our knowledge, this is the first study identifying the factors associated with perceptions on the reasonableness of costs associated with activities implemented to prevent the spread of COVID-19, and the degree to which these costs should be borne by society. Data were taken from a nationally representative online sample. However, future research is required to investigate the attitudes of older individuals aged 75 years and older. Additionally, due to the nature of an online sample, a response rate could not be calculated. Therefore, the possibility of a sample selection bias cannot be entirely dismissed. Moreover, a pretest was conducted showing that, among others, the outcome measures had a high face validity. However, future studies with more sophisticated tools are required to validate our findings. Additionally, only the endpoints were labeled in the dependent variables. However, the middle of the scale was the most frequently chosen response option. It remains unclear whether the respondents selected this option to express neutrality, indifference, did not have an opinion or did not know. In these questions, terms such as “adequacy”, “the objective pursued” or “depending on income” (e.g., linear or progressive) were not further defined. Furthermore, this is a cross-sectional study, and therefore has well-known limitations (e.g., limitations with regard to causality).

## 5. Conclusion

This study identified factors associated with the attitudes of the general population towards the reasonableness of economic costs associated with interventions implemented to prevent the spread of COVID-19, as well as towards bearing these costs as a society. This in turn gives us a rough approximation of the acceptance within society of implemented measures to prevent the spread of COVID-19. This study may ultimately assist in maintaining social cohesion in the general population. Differences in population subgroup perceptions have to be confirmed and should be acknowledged when communicating about costs and cost sharing. Future research in other countries is required to make cross-country-comparisons.

## Supporting information

S1 Table Affect: COVID-19 infection. (DOCX)
