# Supplementary material for: Attitudes towards the economic costs associated with measures against the spread of COVID-19: Population perceptions from repeated cross-sectional data of the nationally representative COVID-19 Snapshot Monitoring in Germany (COSMO)
Source: PLoS One. 2021 Nov 5;16(11):e0259451. doi: 10.1371/journal.pone.0259451 (PMC8570483; doi:10.1371/journal.pone.0259451)
Supplement: S1 Table — (DOCX) [file pone.0259451.s001.docx]

**Supplementary Table 1. Affect: COVID-19 infection**

| **Please select one answer option per line.**  **For me, the new type of corona virus is ...** | | |
| --- | --- | --- |
| Near | O O O O O O O | Far away* |
| Spreading slowly | O O O O O O O | Spreading quickly |
| Something I keep thinking about | O O O O O O O | Something I almost never think about* |
| Scary | O O O O O O O | Not scary* |
| Inflated in the media | O O O O O O O | Not given enough attention in the media |
| Concerning | O O O O O O O | Not concerning* |
| Something I feel helpless about | O O O O O O O | Something I can actively do something about* |

* recoded items
